# Depression and anxiety mediate the relationship between illness representations and perceived distress in patients with chronic pain

**DOI:** 10.1038/s41598-023-42156-4

**Published:** 2023-09-19

**Authors:** Caroline Rometsch, Martin Teufel, Eva-Maria Skoda, Adam Schweda, Fiammetta Cosci, Stephan Zipfel, Andreas Stengel, Christel Salewski

**Affiliations:** 1 Department of Psychosomatic Medicine and Psychotherapy, University Hospital Tübingen, Tübingen, Germany; 2 Clinic for Psychosomatic Medicine and Psychotherapy, LVR University-Hospital Essen, University of Duisburg-Essen, Essen, Germany; 3 Department of Psychology, University of Hagen, Hagen, Germany; 4 Department of Experimental and Clinical Medicine, University of Florence, Florence, Italy; 5 Department of Health Sciences, University of Florence, Florence, Italy; 6 German Center for Mental Health (DZPG), Site Tübingen, Tübingen, Germany; 7 Charité Center for Internal Medicine and Dermatology, Department for Psychosomatic Medicine, Charité - Universitätsmedizin Berlin, Corporate Member of Freie Universität Berlin, Humboldt-Universität zu Berlin and Berlin Institute of Health, Berlin, Germany

**Keywords:** Psychology, Human behaviour

## Abstract

Illness representations explain the individual’s perception and processing of health-related information. In a chronic condition such as persistent pain, illness representations might influence treatment adherence and outcome. This study aims to exploratively identify illness representations of patients with chronic pain and their association to mental disorders and subjective distress. 95 participants admitted to an inpatient university clinic were included. Validated instruments were used to assess illness representations (IPQ-R), mental health disorders (PHQ-D), and subjective distress (PSQ). Sociodemographic data and scores for the instruments were first inspected descriptively. Correlation, regression, and mediator analyses were conducted. Analyses indicated that the distributions of the IPQ-R range toward higher values. In regard to mental disorders (PHQ-D) and subjective distress (PSQ), we found several significant correlations with subscales of the IPQ-R. A regression analysis showed the IPQ-R subscales personal control, emotional representation and sex (males) to be significant predictors of subjective distress measured with the PSQ (F_(11,86)_ = 11.55, *p* < .001, adjusted *R*^2^ = 0.545). Depression, anxiety, and stress syndromes (PHQ-D) significantly mediated the positive association between emotional representations (IPQ-R, predictor) and subjective distress (PSQ, outcome) with a total effect of *c* = .005, 95% CI [.005; .129]. Illness representations play a significant role in evaluating patients’ subjective distress and mental health. It is advised to incorporate illness representations into standard protocols for psychological interventions to comprehend their influence on targeted therapeutic strategies, particularly those tailored for pain management.

## Introduction

Each individuum forms illness representations based on his/her knowledge and ideas about an illness^1^. A conceptual framework for illness representations is the Leventhal’s common-sense model of self-regulation of health and illness (CSM)^2^, that examines the perceptual, behavioral and cognitive processes of an individual facing illness-related threats^3^ and serves as a useful framework for the investigation of illness representations. According to the CSM, illness representations consist of beliefs about the illness identity (such as the number of symptoms), the timeline of the illness (chronic or cyclical), the consequences of a condition, the perceptions of control (personal and treatment control), the coherence or comprehensibility of an illness, and emotional representations (such as fear or anger).

Previous studies on illness representations in chronic conditions showed that illness representations influence the individual’s disease-related self-regulation^1^ and, thereby, act on illness behaviors and treatment options^4,5^. Some clusters of illness representations are associated with a favorable health outcome^6^. In case of a chronic illness condition, in particular treatment control beliefs are associated with a better self-management^7^. For mental illnesses such as psychoses, eating disorders, and depression it was found that illness representations are an indicator for psychosocial well-being^8,9^. Additionally, findings indicate that patients suffering from psychiatric disorders consider their mental constitution as cyclical and chronic^10^. Patients are, understandably, afraid of serious negative consequences^8^. This, corroborated by the literature, emphasizes illness representations being associated with treatment outcomes, and that, in particular, the perception of fewer negative consequences, symptoms and negative emotions as well as a stable course of the illness are associated with favorable outcomes^6^. Furthermore, feelings of control and chronicity are also predictive for outcomes^11^. Even though highly relevant in chronic diseases, there is a lack of knowledge considering the understanding of illness perception in patients with pain disorders.

Chronic pain is defined as a condition with a symptom duration of more than six months^12^. It is a complex disorder with prevalence rates up to 33% worldwide^13^. In addition, chronic pain occurs regularly as a comorbidity with mental health disorders such as depression and anxiety^14,15^, therefore it is a multi-layered disease, which requires a precise understanding of the cognitive processes such as illness representations. Stress is an equally complex construct, which is becoming increasingly relevant in the pathogenesis of chronic pain^16^. However, stress as a concept remains somewhat vague in the literature with undifferentiated combinations of symptoms^17^.

In recent decades, the definition of stress has taken into consideration the subjective reactions to external events or demands^18^, with a focus on the individual’s subjective perception and emotional response^19,20^. The relationship between illness representations and stress has been investigated showing that illness representations are potentially distressing^21^. Several studies have concluded that somatic illnesses such as dermatological symptoms cause severe psychological distress^22^. Further, numerous studies have aimed to identify predictors for subjective distress, e.g., in patients with cancer with several illness-specific variables predicting psychological distress, a history of mental health problems was identified^23^. In cardiovascular conditions, illness representations were found to correlate with psychological distress^24^. Psychological distress has also been investigated as a main outcome variable during the COVID-19 pandemic, where a recent study found anxiety and further anxious attachment as a significant predictor of distress^25^. In conditions of chronic pain, illness presentations account for a significant proportion of the variance in anxiety^26^. Despite finding moderate to large effect sizes for the relations between illness representations and health outcomes, significant heterogeneity suggests potential moderators, including diagnostic, prognostic, and treatment-related variables^27^. Yet, the intricate relationship between illness representations and subjective distress in patients with chronic pain remains underexplored. This study seeks to exploratively investigate this relationship, also considering potential comorbidities with mental illnesses.

In summary, the objectives of the present study are:

1. A comprehensive and explorative assessment of illness representations in patients diagnosed with chronic pain according to the International Classification of Diseases (ICD-10)^28^,
2. An additional explorative assessment of mental comorbidities such as depression and anxiety disorders, and furthermore subjective distress reported by patients with chronic pain,
3. An explorative in-depth analysis of the mutual relationship between the variables, focusing on predictors of subjective distress as well as the identification of mediators related to this association.

## Methods

### Study design and ethical considerations

The cross-sectional study was conducted with patients who suffered from somatoform chronic pain disorders. They were recruited at the Department for Psychosomatic Medicine and Psychotherapy at the University of Tübingen. Patients were asked to participate voluntarily in the study at the beginning of an inpatient multimodal pain treatment. To minimize a potential selection bias, we invited the participants via letters to ensure that all eligible participants can voluntarily participate in the study.

The study was approved by the Ethics Committee of the Medical Faculty of the University Tübingen (765/2015BO2). Participants were informed through written information and a verbal briefing, and they gave informed written consent to participate in the study. The study was performed in accordance with the Declaration of Helsinki^29^.

### Data collection and study methods

Ninety-five patients completed a paper-and-pencil survey (response rate of 70%, 28 patients refused participation). Inclusion criteria were an age over 18 years and having been diagnosed with a chronic pain disorder according to the somatoform disorders of the ICD-10^28^. Exclusion criteria were a current oncological disease, pregnancy or breastfeeding, misuse of drugs or medications, and/or untreated psychotic illness. After a short verbal briefing and obtaining participants’ written consent, the principal investigator or the physician/therapist of the inpatient department handed out the patient survey. Filling in the questionnaires took 30–45 min. The participants’ sociodemographic data were recorded separately from the scores of the psychometric instruments. Sociodemographic data included sex, age, and history of substance use/abuse/addiction (alcohol, nicotine and drugs).

### Assessment instruments

Validated questionnaires in German language were used to assess patients’ illness representations, mental health disorders, and subjective distress.

### Revised Illness Perception Questionnaire (IPQ-R)

The revised Illness Perception Questionnaire (IPQ-R)^30^ is a self-report questionnaire to measure the components of illness representations according to Leventhal’s CSM^3,31^. With 78 items, the German version of the IPQ-R aims to assess the individual’s beliefs and feelings about her or his illness on a five-point Likert scale^32^ with the domains: (1) identity a (patient’s experience of a symptom while being ill) and identity b (patient’s beliefs that a symptom is caused through the illness); (2) timeline; (3) cyclical timeline; (4) consequences; (5) personal control; (6) treatment control; (7) coherence; and (8) emotional representation^30,33^. The items are phrased as such: “*My illness greatly affects my life*” (domain: consequences), “*I believe my illness will persist for a long duration*” (domain: timeline), and “*Thinking about my illness makes me feel depressed*” (domain: emotional representation). High scores on the domains of identity, consequences and timeline represent strongly held beliefs about the number of symptoms; they also represent the occurrence of negative consequences and a high chronicity and cyclical nature of the illness. High scores on the domains of personal and treatment control and coherence represent positive beliefs about control and one’s personal understanding of the illness^31^. In the German version of the IPQ-R, we replaced the word “illness” with “chronic pain” to address, in particular, participants’ chronic pain conditions, as recommended by Moss-Morris^30^. Cronbach’s alpha for the study sample was good (α = 0.80).

### PRIME-MD patient health questionnaire (PHQ)

We used the PHQ-D^34^, which is a brief self-report instrument to assess mental health syndromes. We assessed somatoform syndromes, major depression syndromes, and panic syndromes as well as other anxiety and stress syndromes of the PHQ-D. Somatoform syndromes are assessed with 15 items; the score range is 0–30. Scores over 10 points indicate a moderate expression and over 15 points a severe somatization. The maximal sum score for depression is 27 points. The cut-off is 5 points; a score of 5–10 indicates minor depression and over 10 points major depression. Anxiety syndromes are assessed with scores of 0–21 points; scores over 5 points indicate light symptomatic anxiety, over 10 points moderate symptomatic anxiety, and over 15 points a severe anxiety syndrome. Stress syndromes are assessed with 10 items with a score of 0–20. Scores over 10 points indicate moderate stress syndromes and over 15 points severe stress syndromes. Cronbach’s alpha for the study sample was good (α = 0.80).

### Perceived stress questionnaire (PSQ-20)

The PSQ-20 is a revised version of the Perceived Stress Questionnaire^20^. With 20 items, the PSQ-20 is a self-report assessment instrument to identify subjective perception of psychological distress on a four-point Likert scale^20^. Psychological distress is understood as a representation of subjective stress experiences and is assessed independently of coping strategies. In the analyses, scores are transformed to values 0–100 in four subscales: worries, tension, joy, and demands^19,35^. Worries, tension, and joy represent the participant’s internal stress response. Demands represent the participant’s representation of external stress^19,35^. A total sum score can be transformed, a score over 13 indicates a pathology^19,35^.

### Statistical analyses

For statistical analysis Rstudio (Version 4.3.0)^36^ was used. Sociodemographic data were analyzed descriptively (median, quartiles, range). Since the data did not follow normal distribution^37,38^, we used non-parametric tests for our analyses. We applied chi-square tests for categorical variables to calculate differences between groups. Mann–Whitney U tests were conducted for group comparisons with interval scaled variables. We used a significance level of *p* < 0.05 and a listwise deletion. Alongside the descriptive assessment of the psychometric instruments, multiple linear regression models were employed. Before running the analyses, we tested the assumptions of linear regression, e.g., independence was verified with the Durbin-Watson test (resulting in 1.97, *p* = 0.814, indicating no significant violation), and multicollinearity was checked with the Variance Inflation Factor (VIF) (all VIF values were below of 5). The linear regression with 95% confidence intervals was facilitated via R's *lm()* function to decipher the impact of predictors on the psqsum score. To bolster our findings' reliability, we utilized the *boot()* function from the boot package, applying bootstrapping with 2000 resamples to estimate coefficient distributions^39^ as well as mediator models^40^, while evaluating coefficient bias and standard error. Out of a total of 98 cases (85 valid + 13 missing from PSQ sum score), 18 cases were excluded from the multiple regression modeling due to listwise deletion (amounts to 18.4%). In the process of data evaluation, we employed Little's MCAR test^41^ to assess the randomness of missing data in our dataset. The results (χ_(31)_^2^ = 55.415, *p* = 0.005) indicate that the data are not missing completely at random. Multiple Imputation by Chained Equations (MICE) was applied to handle missing data, using the *mice* package in R. An ANOVA-power analysis was conducted to determine the sample size required in RStudio using the *pwr* package (effect size of 0.2, a significance level of 0.05, and a desired power of 0.8). A multiple regression analysis within a structural equation modeling (SEM) framework was carried out applying a rank-based inverse normal (RIN) transformation^42,43^ using the *lavaan* package^44^. Based on the results of the multiple regression analysis, a mediation analysis was conducted using the *lavaan* package. The objective was to investigate the mediating effect of four variables of the PHQ (anxiety, stress, somatization, and depression) on the relationship between emotional representation and subjective distress. The model was estimated using Maximum Likelihood (ML) with 5,000 bootstraps to acquire more robust standard errors^40^.

### Ethics approval

The study was approved by the Ethics Committee of the Medical Faculty of the University Tübingen (765/2015BO2).

### Consent to participate

Patients were asked to participate voluntarily in the study at the beginning of an inpatient multimodal pain treatment.

## Results

### Sociodemographics

A total sample of *N* = 95 participants were included in our analysis (response rate of 70%). The majority was female (*N* = 75; 78.9%) with a mean age of 46.4 years. Twenty-eight patients opted not to participate, citing reasons related to their current mental well-being and the severity of their pain symptoms. See Table 1 for further information.

**Table 1:** Sociodemographic data for the chronic pain sample (N = 95).

Sample description (*N* = 95)			
Age (years) (*N* = 95)			
	Median	49.0	
	Quartile 1	37.0	
	Quartile 3	56.0	
	Range	18–80	
Sex (*N* = 95)		Total	Percentage
	Female	75	78.1%
	Male	20	21.1%
Drug use		Total	Percentage
Smokers (*N* = 94)			
	Never smoked	23	24.5%
	Stopped smoking	30	31.9%
	Smoke sometimes	17	18.1%
	Smoke regularly	24	25.5%
Alcohol consumption (*N* = 95)	No	86	89.6%
Caffeine consumption (*N* = 95)	None	8	8.4%
	1–3 cups/day	68	71.6%
	More than 3 cups/day	21	20.0%

### Illness representations

**Figure 1:**
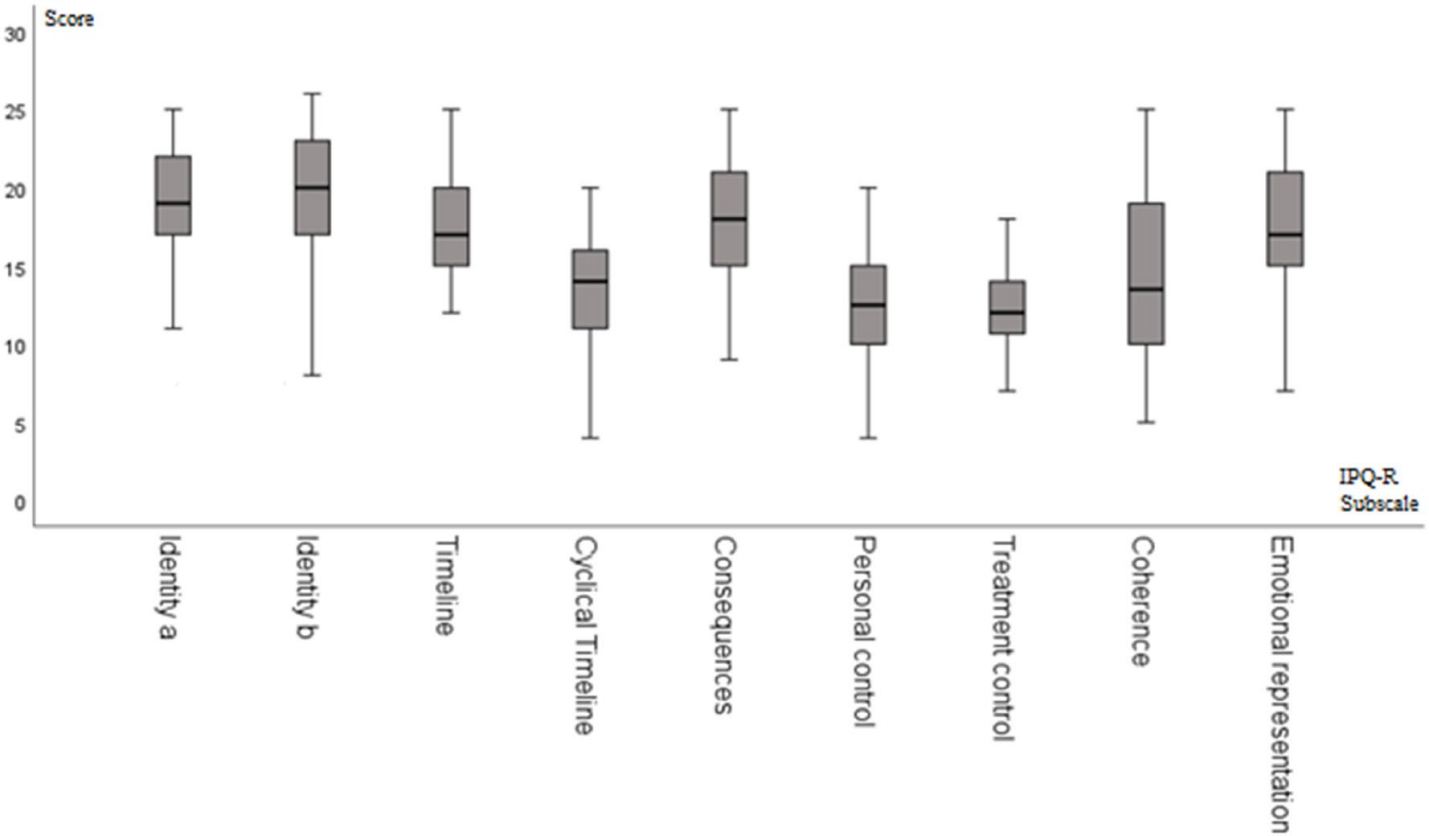
Distribution of the subscales of the IPQ-R with medians, quartiles, minimum and maximum.

The results of the domains of the IPQ-R are presented as medians and quartiles as well as minimum and maximum (Fig. 1). We found high scores in the IPQ-R subscales of timeline, cyclical timeline and emotional representation and low values for personal and treatment control and coherence.

### Mental comorbidities (PHQ-D)

**Figure 2:**
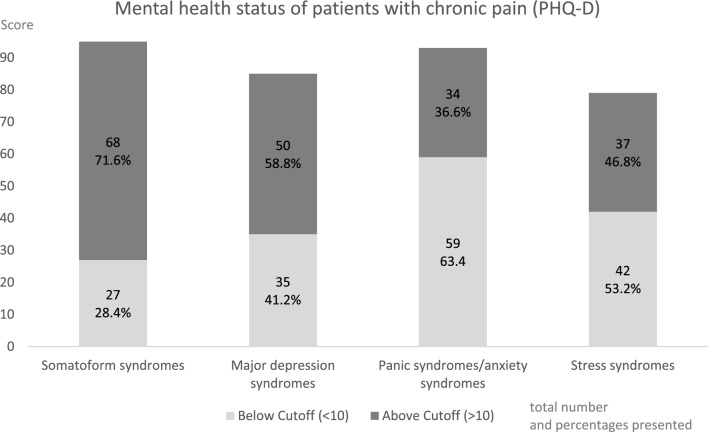
Results of the PHQ-D for each subscale with the total number and percentage above and below the cutoff (10 points) for somatoform syndromes, major depression syndromes, panic syndromes/anxiety, and stress syndromes.

We found a median for somatoform syndromes of 13.0 (*Q1: 9.0, Q3: 16.0, range 3–24);* for depression syndromes, the median was 11.0 (Quartile 1 (*Q1): 7.0,* Quartile 3 (*Q3): 18.0, range 2–26).* Panic syndromes/anxiety syndromes were calculated with a median of 7.0 (*Q1: 5.0, Q3: 11.0, range 0–18)* and stress syndromes with a median of 9.0 (*Q1: 6.0, Q3: 12.0, range 1–14).* Figure 2 shows the patients (total number and in percentage) who exceeded the cutoff of 10 points, which indicates the presence of a specific mental disorder.

### Perceived subjective stress (PSQ)

In our sample, the sum score yielded a median of 65.0 (*Q1: 44.2, Q3: 80.0, range 8–97)*. The median scores for the subscales were 60.0 for worries (*Q1: 40.0, Q3: 80.0, range 0–100)*, 80.0 for tension (*Q1: 56.7, Q3: 86.7, range 7–100)*, 26.7 for joy (*Q1: 13.3, Q3: 46.7, range 0–87)*, and 46.7 for demands (*Q1: 23.3, Q3: 66.7, range 0–100)*. For further understanding, we calculated the results regarding the definition for average, above average, and below average based on the mean average scores of healthy adults (*M* = 33, *SD* = 17^19, 35^, Table 2).

**Table 2:** Results of the PSQ-20 subscales in percentage. The definitions for above average, average, and below average values were adopted from Fliege^19^.

	Worries (%)	Tension (%)	Joy (%)	(%)
Above average	66.3	74.7	1.1	41.1
Average	32.6	24.2	30.5	37.9
Below average	1.1	1.1	68.4	21.1

### Correlations analysis

#### Illness representations and mental comorbidities (IPQ-R and PHQ-D)

**Figure 3:**
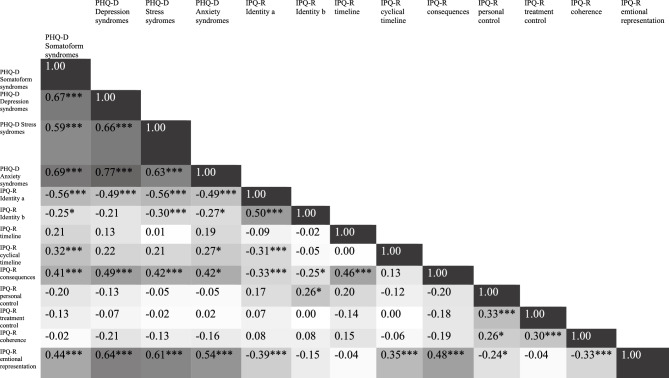
Spearman-Correlation between the subscales of IPQ-R and the subscales of the PHQ-D with darker color indicating a higher correlation. *Note.* Significance * *p* < .05, ** *p* < .01, *** *p* < .001.

Figure 3 shows the correlations between the IPQ-R and PHQ-D. In particular, the analyses showed significant correlations for IPQ-R-scale emotional representation with PHQ-D somatoform syndromes ρ(83) = 0.44 *p* < 0.001, PHQ-D depression syndromes ρ(83) = 0.64 *p* < 0.001, PHQ-D anxiety syndromes ρ(77) = 0.61 *p* < 0.001, and PHQ-D stress syndromes ρ(81) = 0.54 *p* < 0.001.

#### Multiple linear regression

A multiple linear regression was calculated to identify predictors for patients’ subjective distress using the PSQ sum score as a dependent variable. The entire predictive value of the regression model reached significance (F_(11,86)_ = 11.55, *p* < 0.001) with adjusted *R*^2^ = 0.545. Significant predictors for the subjective distress measured by the PSQ sum score were the IPQ-R subscales personal control, emotional representation, and sex (male, Table 3).

**Table 3:** Multiple linear regression with bootstrap to identify predictors for the PSQ sum score with significant results for the independent variables of the IPQ subscales personal control, emotional representation as well as sex (males).

Variables	b	SE b	BCa-95% CI	*p*-value (bootstrap)
Constant	− .050	.19	(− .438; .337)	.797
Identity a	− .005	.004	(− .012; .003)	.252
Identity b	− .003	.003	(− .010; .003)	.325
Timeline	.004	.006	(− .010; .015)	.543
Cyclical timeline	− .007	.005	(− .018; .004)	.216
Consequences	.010	.006	(− .010; .022)	.097
Personal control	.012	.005	(.002; .022)	.023*
Treatment control	− .005	− .008	(− .022; .012)	.541
Coherence	.001	.004	(− .010; .009)	.754
Emotional representation	.028	.004	(.019; .037)	< .001***
Age	.002	.001	(− .0003; .004)	.091
Sex	.002	.039	(− .0003; .004)	.002**

*R*^2^ = 0.545. Significance * *p* < .05, ** *p* < .01, *** *p* < .001, Bca = Bias corrected and accelerated confidence interval.

#### Mediation analysis

**Figure 4:**
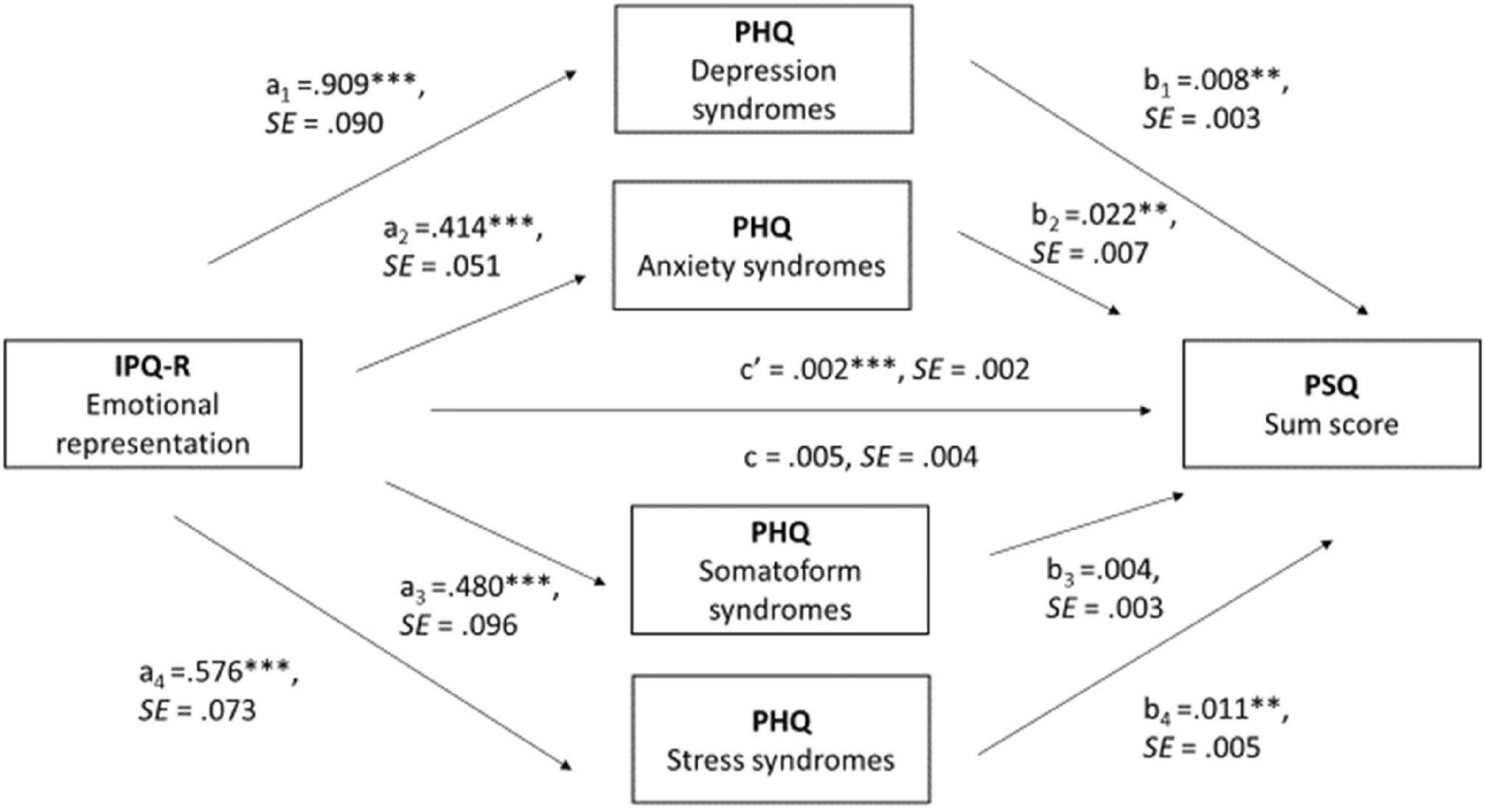
Results of the mediator analyses. Mediation model with IPQ-R (predictor), PHQ depression syndromes, PHQ anxiety syndromes, PHQ stress syndromes, and PHQ somatization (mediators), PSQ sum score (outcome). *Notes.* c = path of predictor to outcome, without inclusion of mediator (total effect); a_1/2/3/4_ = path of predictor to mediators; b_1/2/3/4_ = path of mediators to outcome; c’ = path of predictor to outcome including mediator (total indirect effect). Significance * *p* < .05, ** *p* < .01, *** *p* < .001.

Based on the findings from the regression analysis, we sought to further elucidate the emotion-based associations, in particular the relationship between emotional representation and subjective distress. Consequently, affective disorders assessed using the PHQ were incorporated as mediating variables (Fig. 4).

The mediation analysis (see Fig. 4) indicated that emotional representation had a nonsignificant direct effect on subjective distress with an estimate of 0.005 (*p* = .181) and indirect effects of depression, anxiety, and stress syndromes which significantly mediated the relationship between emotional representation and subjective distress, with standardized indirect effect sizes of 0.218, 0.147, and 0.185, respectively. Somatization's mediation effect was not significant (standardized indirect effect = 0.053, *p* = 0.168).

## Discussion

In this study we investigated illness representations, mental comorbidities and subjective distress in a sample of patients with chronic somatoform pain, as well as possible relations between these variables. Regarding illness representations, patients with chronic pain reported high scores in the IPQ-R subscales timeline, cyclical timeline and emotional representation and low scores for personal and treatment control and coherence. Illness representations correlated with mental comorbidities assessed with the PHQ-D. The subsequent linear regression analysis showed significant results for the IPQ-R subscales personal control, emotional representation and male sex as predictors for perceived subjective distress. Moreover, depression, anxiety, and stress syndromes were significant mediators in the positive association between emotional representation (as one dimension of illness representations) and perceived subjective distress.

A recently published study on chronic widespread pain showed that patients believed their pain to be long-lasting and to affect their emotional well-being^45^. Further, patients suffered from fears of having negative consequences in their lives^45^. This matches the results of the present study with regard to the elevated scores for the IPQ-R subscales timeline, cyclical timeline and emotional representation. This may indicate that the participants of our chronic pain sample understand their illness as a chronic condition with low variability and high negative emotions. Another study on patients with chronic headache underpins our findings, with beliefs of a long-lasting illness, associated with negative life consequences and emotional distress^46^. Low values for personal and treatment control and coherence in our distribution analysis could be interpreted as feelings of lack of control over their chronic pain disorder, but also a lack of control for treatment options. Such perceptions may arise when patients do not fully grasp the complexities associated with their chronic pain disorder, potentially leading to challenges in their therapeutic journey. Similar findings are described in a sample of patients with headache with insufficient feelings of control; patients experienced less control and illness coherence in the sense of understanding their illness^46^.

Beyond conditions related to pain, individuals with psychosis, bipolar disorder, eating disorders, and depression often struggle to recognize or accept the long-term nature of their mental disorder^8^. Additionally, patients consider their mental disorders as having serious negative consequences^8^. Illness representations with a leading conviction of a chronicity and without control was associated negatively with coping and help-seeking strategies as well as treatment adherence^8^. With regard to illness behavior, similar results were found for patients with chronic pain, such as headache^46^ or pelvic pain^47^. In summary, our study distinctively illustrates that chronicity, a lack of control, and pronounced negative consequences are core illness representations in patients with chronic pain. While this aligns with findings from previous studies on other mental disorders and pain conditions, it stands out as a novel insight within the domain of somatoform disorders in a psychosomatic context.

In contrast to other approaches, the theoretical frame of the Leventhal’s common-sense model of self-regulation of health and illness provides an explanatory model that integrates these results. Furthermore, the CSM is also a useful framework for translating the study results into clinical practice. With a focus on patients with chronic pain, researchers found that illness beliefs about the serious consequences of suffering from pain are important predictors of treatment outcomes^11^. However, there are findings pointing to the relevance of inter-individual variability in illness and treatment perceptions explained by disease-related variables, but also by patients’ personal characteristics and interaction experiences^48^. Nevertheless, our study highlights how essential the elicitation of illness representations is for planning and directing appropriate pain-specific treatment.

Literature reports a negative correlation between depression, measured with the Beck Depression Inventory (BDI), and the IPQ-R subscales consequences and emotional representation^49^. This is in line with our analysis, resulting in significant negative correlations between depression syndromes and the IPQ-R identity as well as significant positive correlation with IPQ-R consequences and emotional representation. Furthermore, depression seems to be associated with less control^50^. Similar results are described in the literature regarding anxiety and its associations with illness concepts and negative consequences for life and higher concerns in patients with chronic, rare diseases^50^. Studies on inflammatory bowel diseases found dysfunctional illness representations in the IPQ-R subscales of consequences, personal control, emotional representations, and treatment control^51^. For patients with chronic pain conditions, research indicates that the stronger their belief in facing adverse outcomes, the higher their reported levels of anxiety and depression^45^, thereby emphasizing the role of illness representation in the outcome of mental well-being. These results align with our findings, stressing the significance of frequently co-occurring depression in patients with chronic pain. This underscores the need to consider both the patients' illness beliefs and the reciprocal influence these beliefs might have on the onset of mental co-morbidities.

Illness representations gain prominence due to their fundamental link with subjective distress. One study systematically reviewed a total of 1050 articles and showed that illness perceptions explained 25–30% of variance in patients’ emotional health outcomes^52^. In particular, the perceptions of consequences of the illness and the emotional representations had the strongest relationship with the outcomes. The authors emphasized the importance of including emotional representations as a predictor for health outcomes as they have a strong association with anxiety^52^. We conducted a multiple linear regression analysis with the IPQ-R subscales of personal control and emotional representation (a dimension of illness representations) as well as sex (males) as predictors for perceived subjective distress in chronic pain patients. The results indicated a significant regression equation, suggesting that both personal control and emotional representation are significant predictors of subjective distress. Specifically, patients with higher scores on personal control and emotional representation tend to report higher subjective distress. In mental health studies, a strong sense of personal control is crucial. A robust sense of personal enhances an individual's capacity to mitigate adverse events and manage their repercussions effectively^53^. In particular in pain patients a low control sense was associated with depression, anxiety, and distress^54^. However, results indicate a higher subjective distress in case of a higher sense of control in this study. This discrepancy suggests that the relationship between personal control and psychological distress may be more complex than previously understood, possibly varying based on context or specific conditions which have to be investigated in future studies. Additionally, the analysis indicated that, on average, males report less subjective distress than females. Particularly, the association with the male sex is notable and in line with literature which commonly links female sex with subjective distress due to a heightened reporting of somatic symptoms^55^. Further, in contrast to the systematic review^52^, the subscale consequences did not turn out to be significant in our analysis. There is evidence that the IPQ-R as a questionnaire shows questionable confounding between the domains of emotional representation and consequences^56, 57^, which may explain the fact that we did not find consequences to be a significant variable in the equation. Despite, further investigations are essential to find a clear answer for this inconsistency. To get a deeper understanding of the relationship between emotional representation and subjective distress, we found depression, anxiety, and stress syndromes to be significant mediators. This indicates that the influence of illness representations on subjective distress might be channeled through these emotional and psychological states. Notably, somatoform syndromes did not significantly mediate the relationship, suggesting it may not play a critical role in this context. Strong associations between emotional representation and anxiety are described in literature^58^, hence, we conclude that patients with a shorter illness duration of chronic pain and lower emotional burdens suffer from less psychological distress, have a better quality of life, and are in better mental constitution than patients with a high chronic pain identification, less control beliefs, and more negative emotions. However, attention should be paid to mental illnesses, such as depression or anxiety disorders, which are often comorbid with chronic pain and influence this relationship.

In this cross-sectional study, we aimed to assess illness representations of patients suffering chronic pain. Selection bias may have occurred due to the sample recruitment; participants are patients involved in intensive multimodal pain treatment at the University Clinic of Tübingen. Patients are highly burdened and thus are recommended for intensive pain treatment. Their responses could be biased since they were investigated prior to an intensive multimodal pain treatment, and they were waiting for be administered to the clinic for receiving treatment. Further on, according to the power analysis, a sample size of approximately 393 participants would be needed. However, due to practical constraints, our study included a total of 95 participants, which has to be considered when interpreting these results. The model's goodness-of-fit statistics indicate that the proposed model may not be the best representation. Future studies might consider exploring other potential mediators. Voluntary participation could be another selection bias. Future studies should also recruit a comparable control sample, for instance by using propensity score matching. Although Leventhal postulated that the scores of the CSM dimensions may change over time^59^, there are findings related to irritable bowel syndrome showing that illness representations remain stable in a longitudinal examination^60^. We used a cross-sectional study design, but further investigations of illness representation in a longitudinal study design are called for.

Illness representations of patients with a chronic pain are associated with subjective distress. It is likely that altering illness representation changes the subjective distress in patients with chronic pain. Based on our observations, it is recommended that these findings are integrated into standardized protocols for psychological interventions targeting individuals with chronic pain conditions, thereby optimizing therapeutic strategies and potentially enhancing treatment outcomes for this patient population. Further on, it is advisable to integrate targeted psychoeducation sessions, incorporate cognitive behavioral therapy into treatment regimens, conduct regular assessments of patients' perceptions and distress levels, adopt a multidisciplinary approach involving both medical and psychological professionals, and facilitate patient support groups to share experiences and coping strategies, always taking illness representations and their association with subjective distress into account. Additionally, mental disorders such as depression, anxiety, and stress syndromes have to be considered as they mediate this relationship between illness representations and subjective distress. Exploring this relationship further in psychotherapy research will likely yield actionable insights for clinician-patient interactions.

## Data Availability

Data are available on request to the corresponding author.

## Abbreviations

IPQ-R: Revised illness perception questionnaire
PHQ-D: PRIME-MD patient health questionnaire
PSQ: Perceived stress questionnaire
Q1: 1. Quartile
Q2: 2. Quartile
